# Geopolymer Based on Mechanically Activated Air-cooled Blast Furnace Slag

**DOI:** 10.3390/ma13051134

**Published:** 2020-03-04

**Authors:** Ilda Tole, Magdalena Rajczakowska, Abeer Humad, Ankit Kothari, Andrzej Cwirzen

**Affiliations:** Building Materials, Department of Civil, Environmental and Natural Resources Engineering, Luleå University of Technology, 97187 Luleå, Sweden; magdalena.rajczakowska@ltu.se (M.R.); abeer.humad@ltu.se (A.H.); ankit.kothari@ltu.se (A.K.); andrzej.cwirzen@ltu.se (A.C.)

**Keywords:** mechanochemistry, alkali activation, air-cooled slag, ground granulated slag, mechanical activation, cement-free mortars

## Abstract

An efficient solution to increase the sustainability of building materials is to replace Portland cement with alkali-activated materials (AAM). Precursors for those systems are often based on water-cooled ground granulated blast furnace slags (GGBFS). Quenching of blast furnace slag can be done also by air but in that case, the final product is crystalline and with a very low reactivity. The present study aimed to evaluate the cementitious properties of a mechanically activated (MCA) air-cooled blast furnace slag (ACBFS) used as a precursor in sodium silicate alkali-activated systems. The unreactive ACBFS was processed in a planetary ball mill and its cementing performances were compared with an alkali-activated water-cooled GGBFS. Mixes based on mechanically activated ACBFS reached the 7-days compressive strength of 35 MPa and the 28-days compressive strength 45 MPa. The GGBFS-based samples showed generally higher compressive strength values.

## 1. Introduction

The production of Portland cement uses processing temperatures exceeding 1400 °C, thus leading to a large CO_2_ footprint [1]. Environmental benefits can be achieved by the use of binders made of CO_2_ neutral alumina silicate rich industrial by-products or wastes. Fly ash and ground granulated blast furnace slag (GGBFS) are typical examples. The GGBFS, which is in the focus of this research, can be utilized either as partial or full replacement of Portland cement. In the case of partial replacement, the GGBFS undergoes a secondary pozzolanic reaction due to the alkali activation process induced by the hydrating Portland cement [2]. At full replacement, the solidification is controlled by a combination of alkali activation processes and hydration. The chemical reactions are induced by strong alkalis such as sodium silicate or sodium hydroxide [3,4,5,6].

Blast furnace slag (BFS) is a by-product of iron production in a blast furnace. The global production of slag is estimated to reach around 450 million tons per year and the value is expected to increase in the coming years [7]. In the process, a mixture of pelleted iron oxides, limestone and carbon is heated at around 1500 °C. The formed liquid slag is removed from the blast furnace and can be cooled in different ways (e.g., by air-cooling, air quenching, water spraying, water quenching, etc.) [8].

The applied quenching method affects the properties of the produced BFS, especially including its crystallinity. The obtained solid material can be crushed and grinded, producing the GGBFS. The water-cooled GGBFS is highly amorphous, has strong pozzolanic properties and good reactivity in alkali-activated systems. Unfortunately, the demand for large amounts of water during quenching produces pollution and negatively affects the environment [9].

An alternative quenching method based on air-cooling produces a crystalline material, air-cooled blast furnace slag (ACBFS), which is unreactive and is mainly used as coarse aggregate for road bases and concrete production [10,11,12]. Earlier studies showed that mechanical activation is able to induce amorphization and structural disorder through the application of high-energy impact forces during grinding leading to the increased chemical reactivity [13,14]. Different mechanically activated minerals showed significant enhancement of their chemical reactivity [15,16,17,18,19].

The main aim of this study was to verify if ACBFS could be activated by the application of intensive grinding and to produce reactivity similar to the water-cooled BFS.

## 2. Materials and Methods

ACBFS and water-cooled GGBFS were used as precursors for this study. Both materials originated from the same source. Their chemical composition was similar but with a slightly higher amount of the Fe_2_O_3_ in the case of the air-cooled slag (ACBFS) (Table 1).

ACBFS was mechanically processed using a planetary ball mill with a 500 mL capacity. The used ball mill was a Retsch equipment, type PM100 (Retsch GmbH, Haan, Germany), with 12 balls having a diameter of 200 mm. The rotation speed was 500 rpm. Ball to powder ratio of 25 and grinding duration of 20 min were used for all grindings.

Successively, 54 types of mortars were prepared for the analysis. Combinations of various parameters were selected based on the full factorial design of experiments (Table 2). The water to binder (w/b) ratio was 0.45, 0.5 and 0.55. The mass ratio of sand to slag was kept constant at 1:1. All mixes were activated by the liquid sodium silicate (SS) provided by the PQ Corporation (PQ Corporation, Valley Forge, PA, USA). The SS had an alkali modulus (AM, mass ratio SiO_2_/Na_2_O) of 2.2, with 34.37 wt.% of SiO_2_, 15.6 wt.% of Na_2_O and a solid content of 49.97 wt.%. The AM of the SS was adjusted to reach three different values of 1, 1.5 and 2.0 by addition of sodium hydroxide pellets (98% purity). Water contained in the sodium silicate solution was subtracted from the calculated w/b ratio. The alkali activator was added as 10, 15 and 20 wt.% of the binder content calculated as a solid material.

Mortar, produced using only the not-treated ACBFS as a precursor, did not solidified. Therefore, only samples prepared with the mechanically activated ACBFS (MCA-ACFBS) are included in this study.

**Figure 1:**
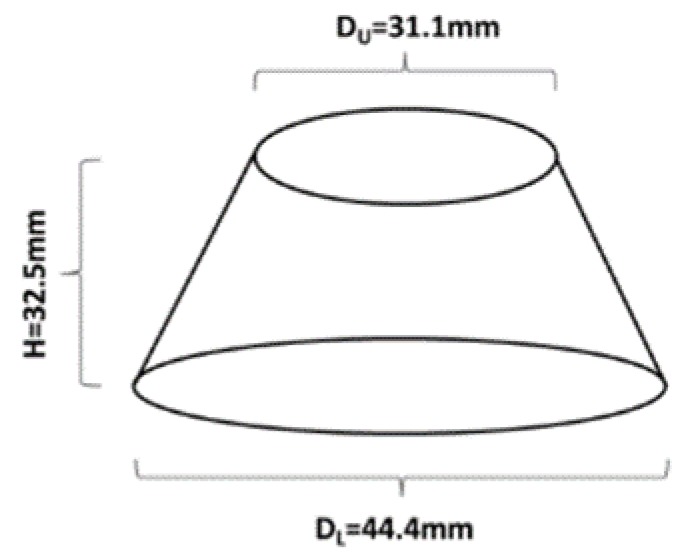
Schematic representation of the mini slump cone used to evaluate the workability.

Workability was determined by means of the in-house developed mini slump flow test. The used cone had the following dimensions d = 31.1, D = 44.4 and h = 32.5 (Figure 1). The slump flow diameter was calculated as an average of two measured values.

**Figure 2:**
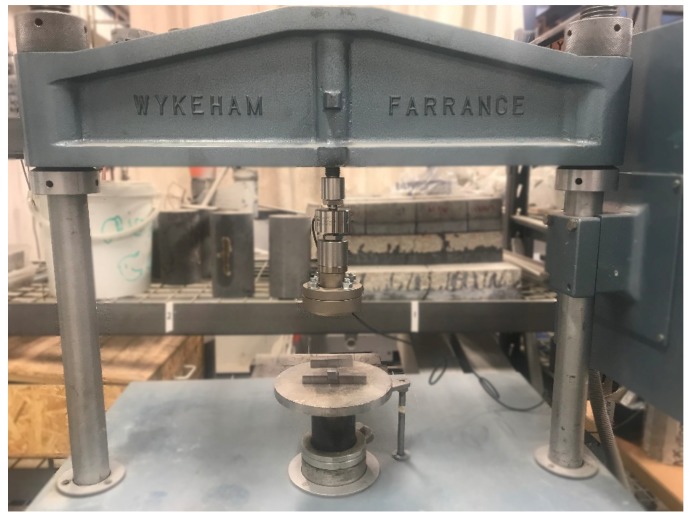
Image of the compressive strength test settings.

All mixes were produced using a small volume vacuum mixer, type Ecovac. Mixing duration was 2 min and the mixing speed was 390 rpm. The test specimens had dimensions of 12 × 12 × 60 mm^3^ and were cast into Teflon molds without application of demolding oil. Three samples were prepared for each mix. After demolding, all samples were stored in plastic boxes exposed to laboratory conditions until testing. The compressive strength was determined after 7 and 28 days, using a CTM test machine combined with the QuantumX MX440B universal measuring amplifier (HBM, Darmstadt, Germany), (Figure 2). The loading speed was 0.5 mm/min. The compressive strength results were calculated as an average of three different measurements.

An X-ray diffractometer type Empyrean from PANalytical with PIXcel 3D detector (Malvern Panalytical Ltd., Royston, UK) was used to determine the chemical phase composition of the solidified mortars. Measurements were done using Cu-K radiation with a wavelength of 1.54060 Å, generated at 45 kV and 40 mA. The step size was 0.0260. The measurements were done from a range of the angle 2θ from 5° to 45°. No pre-treatment and back-loading sample holders were used. Panalytical’s Highscore Plus software, equipped with a COD database, was used to determine the phase composition.

Microstructure of the solidified samples was analyzed using Scanning Electron Microscope, type Jeol JSM-IT100 combined with EDS analyzer, type Bruker (JEOL Nordic AB, Sollentuna, Sweden). Samples were stored for 24 h in isopropanol and subsequently placed in a vacuum chamber and impregnated with a low viscosity epoxy resin. The impregnation was done 7 and 28 days after casting. After curing, samples were grinded and polished using diamond spray containing 9, 3 and 1 µm diamond particles.

All images were obtained using a backscattered electron detector at two magnifications: 1000× for the particle size analysis and 4000× for the phase composition analysis. The following parameters were used: accelerating voltage of 15 kV, accelerating current of 60 µA, low vacuum mode of 60 Pa. The phase composition analysis was performed on 10 images having the magnification of 4000× while the EDS spot analysis was used to collect data from 20 different locations on each image. The analyzed points were chosen manually based on the grey level histogram [20].

The particle size distribution was calculated using SEM images having a spatial resolution of 0.025 µm per pixel. The image processing and analysis was performed with the ImageJ software [21,22,23]. The median filter with 2-pixel kernel was applied to remove the noise followed by an automatic thresholding enabling binarization of images. Powder particles were segmented from the impregnating resin matrix. The analysis of the binarized images included determination of the basic morphological parameters (i.e., Feret diameter, perimeter, and surface area).

## 3. Results and Discussion

The suitability of precursors in respects to their chemical composition for alkali-activated systems can be determined by the alkalinity factor Kb and the so-called hydration modulus (HM). Alkaline and neutral slags are considered to be valuable precursors for alkali-activated materials. Evaluation of the utilized slag alkalinity was calculated as the ratio between the amounts of basic and acid oxides using Equation (1) [24,25,26]. Slags with a Kb equal to 1 were classified as neutral, while Kb > 1 slag was considered basic and Kb < 1 as acidic.

(1) $$K_{b}=\frac{\mathrm{CaO}+\mathrm{MgO}+{\mathrm{Fe}}_{2}O_{3}+K_{2}O+{\mathrm{Na}}_{2}O}{{\mathrm{SiO}}_{2}+{\mathrm{Al}}_{2}O_{3}},$$

The hydration modulus was defined as the ratio between the sum of calcium, magnesium and aluminum oxides and the silicon oxides using Equation (2). Values of the hydration modulus (HM) higher than 1.4 were assumed to indicate efficient hydration during the alkali activation process [27,28].

(2) $$\mathrm{HM}=\frac{\mathrm{CaO}+\mathrm{MgO}+{\mathrm{Al}}_{2}O_{3}}{{\mathrm{SiO}}_{2}},$$

According to the calculated values, the MCA-ACBFS were classified as a basic slag, while the GGBFS were slightly acidic. The hydration modulus was higher than 1.4 indicating good hydration properties. In addition, MCA-ACBFS had a higher hydration modulus than the water cooled GGBFS (Table 3).

**Figure 3:**
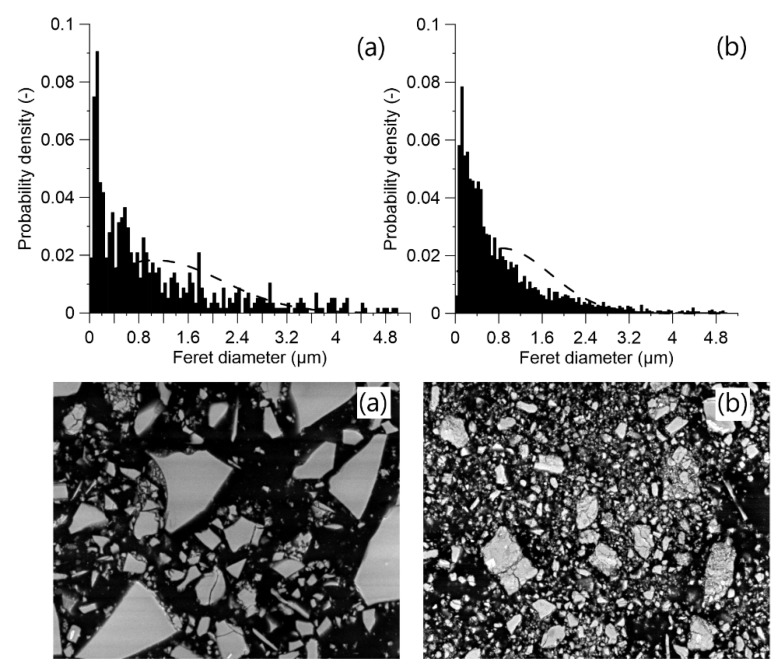
Particle size distribution for: (**a**) water cooled GGBFS and (**b**) MCA-ACBFS before alkali activation.

The applied MCA process on ACBFS resulted in finer particles size distribution in comparison with the water-cooled GGBFS (Figure 3). Fragmentation of ACBFS particles was visible from the obtained SEM micrographs. The average particle size of the GGBFS sample was approximately 1.8 µm, while the average particle size of ACBFS sample after the ball milling (BM) process was only 1 µm.

**Figure 4:**
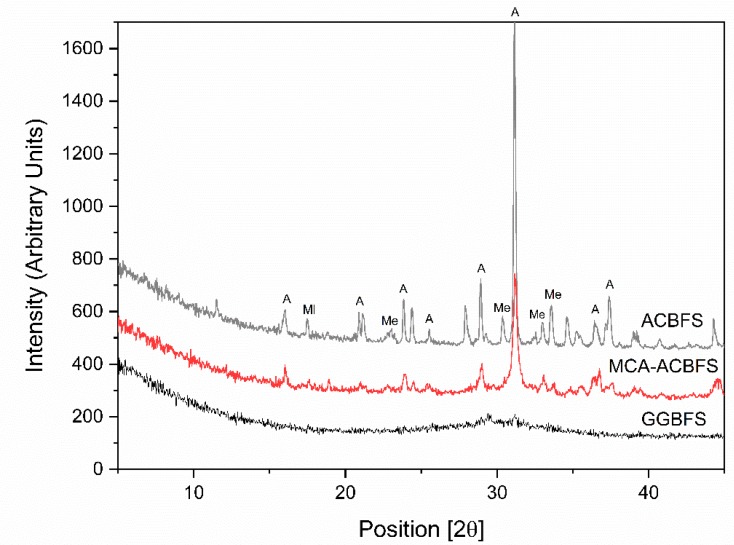
XRD pattern of ACBFS, MCA-ACBFS and GGBFS.

XRD analysis was used to identify changes of the crystallinity after application of the mechanical activation process [29,30]. The XRD diagrams showed significant differences between the ACBFS and the GGBFS as well as MCA-ACBFS (Figure 4). The untreated ACBFS contained crystalline akermanite (A), melilite (Ml) and merwinite (Me). Application of the BM process resulted in a partial destruction of A and Ml phases, visible as decreased intensities and broadened peak areas.

**Figure 5:**
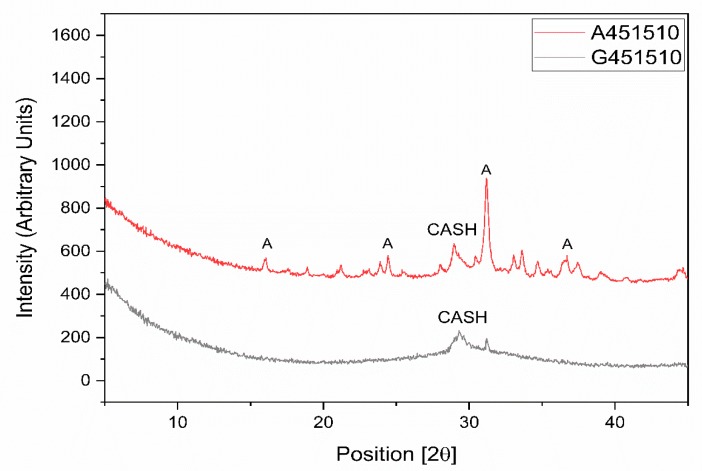
XRD diagrams for sodium silicate activated slags with water to binder (w/b) = 0.45, alkali modulus (AM) = 1.5 and sodium silicate (SS) = 10%.

XRD analysis of the alkali-activated pastes based on GGBFS and MCA-ACBFS revealed a formation of the aluminate-substituted calcium silicate hydrate (CASH) in both cases (Figure 5). The CASH phase is the main phase observed to form in sodium silicate activated GGBFS [31,32]. CASH was detected in XRD for both sodium silicate activated slags (Figure 5). The not-amorphized akermanite (A) phase was still visible after sodium silicate activation of the MCA-ACBFS. Akermanite seemed to be inert to the alkali activation process, thus complying with the latent reactivity of the water-cooled slag before the mechanical activation. Optimization of the grinding parameters could produce higher degree of amorphization for the akermanite (A) phase, providing a final phase composition similar to the GGBFS.

**Figure 6:**
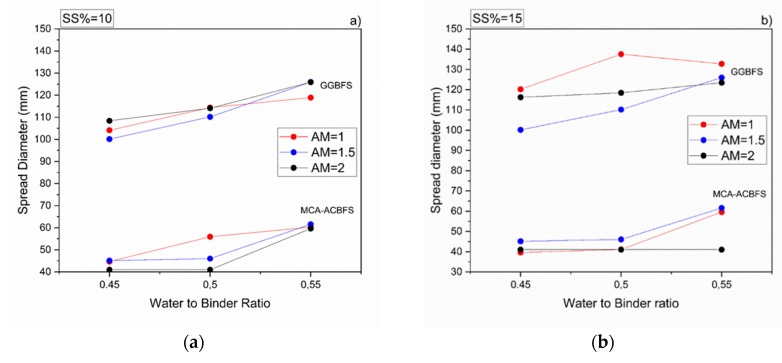

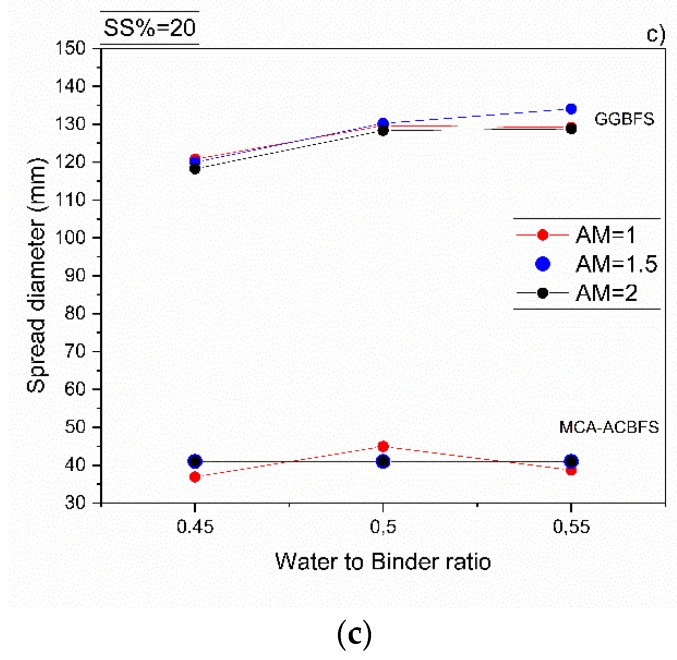
Workability of sodium silicate activated mortars containing MCA-ACBFS and GGBFS as precursors, for different w/b, alkali modulus and solid solution content, respectively. (**a**) 10%, (**b**) 15% and (**c**) 20%.

All mortar samples made from ACBFS and GGBFS, with alkali modulus 1 and solid solution of 10%, showed an acceptable workability. Mortar samples made from MCA-ACBFS presented a lower slump flow than the GGBFS-based samples. It was related to a finer particle size distribution and a higher specific surface area caused by the applied MCA process (Figure 3 and Figure 6) [33]. The increased w/b ratio enhanced the workability of mixes activated with 10 and 15 wt.% of the SS. Mortars activated with 20 wt.% of SS showed a similar workability independently of the used AM.

**Figure 7:**
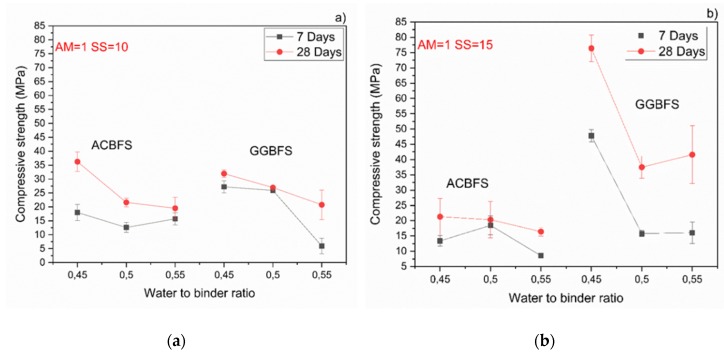

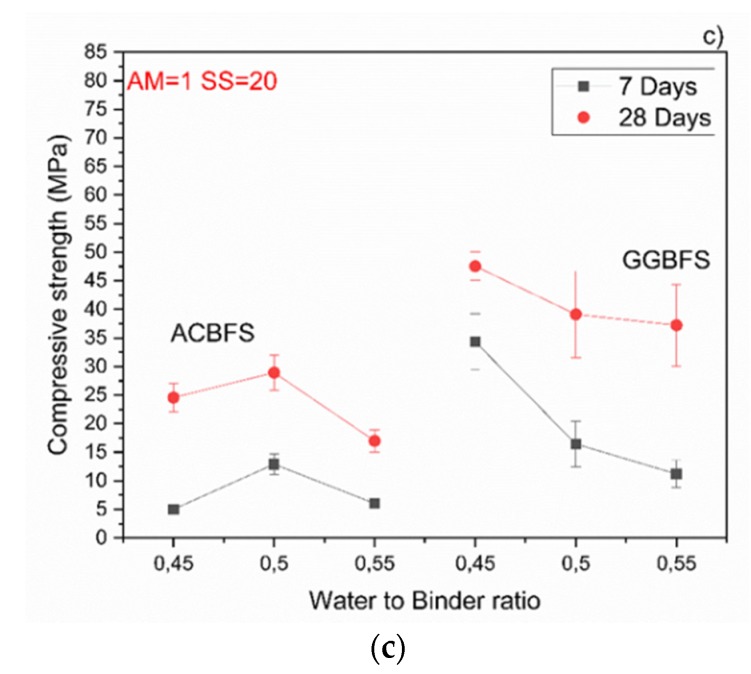
Effects of w/b ratio and SS amount on 7- and 28-days compressive strength values for mixes activated with sodium silicate having Ms = 1 and SS content of; (**a**) 10%; (**b**) 15%; (**c**) 20%.

The MCA-ACBFS based mortars activated with the SS having the alkali modulus of 1 and containing 10 wt.% of the SS alkali activator showed comparable or higher 28-day compressive strength values than the GGBFS (Figure 7). Mortars based on MCA-ACBFS reached 20–40 MPa while those based on the water-cooled GGBFS reached 20–30 MPa. In general, a higher w/b ratio decreased the compressive strength for all tested mortars.

**Figure 8:**
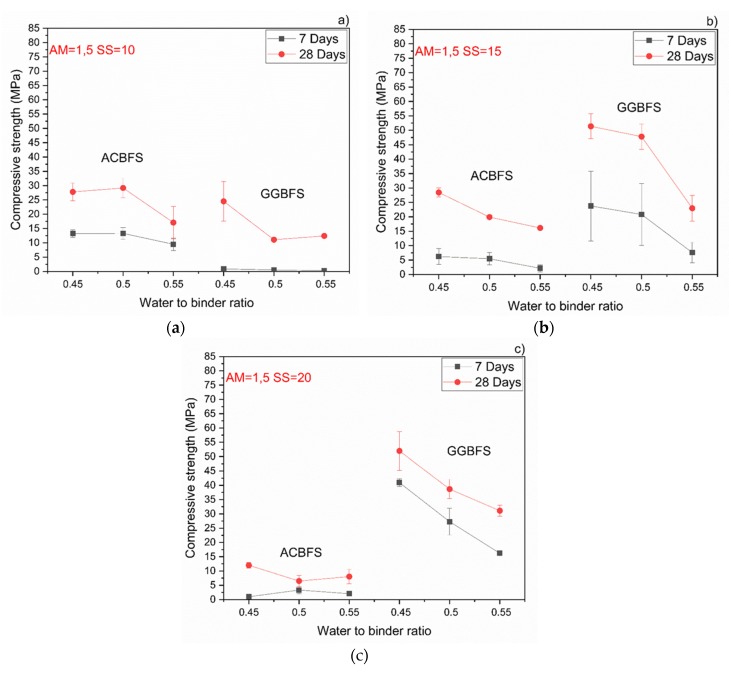
Effects of w/b ratio and SS amount on 7- and 28-days compressive strength values for mixes activated with sodium silicate having Ms = 1,5 and SS content of; (**a**) 10%; (**b**) 15%; (**c**) 20%.

The compressive strength values tended to increase at lower w/b ratios for both types of the used slags. Usually the w/b ratio had a smaller effect on mechanical properties in the case of the AAM and the chemical composition of the precursor was the dominant factor [34]. In this case, however, the initial chemical composition of both slags was very similar. MCA-ACBFS showed decreased compressive strength values at lower w/b ratios. The same trend was observed for higher alkali modulus (Ms = 1.5) and for both analyzed slags. Higher w/b ratio gave decreased strength measured after 7 and 28 days. The higher amount of water presumably increased the binder matrix porosity, which lowered the measured strength. Increasing the alkali modulus to 1.5 strongly influenced the mechanical properties at 7 days for GGBFS samples (Figure 8). Extremely low values were registered accompanied by not fully dried samples with greenie color in the inner part.

The workability of the GGBFS-based concrete was not affected by a higher alkali modulus (Figure 6). On the contrary, mixes based on the MCA-ACBFS, showed a decreased slump flow and a rapid setting at higher AM.

**Figure 9:**
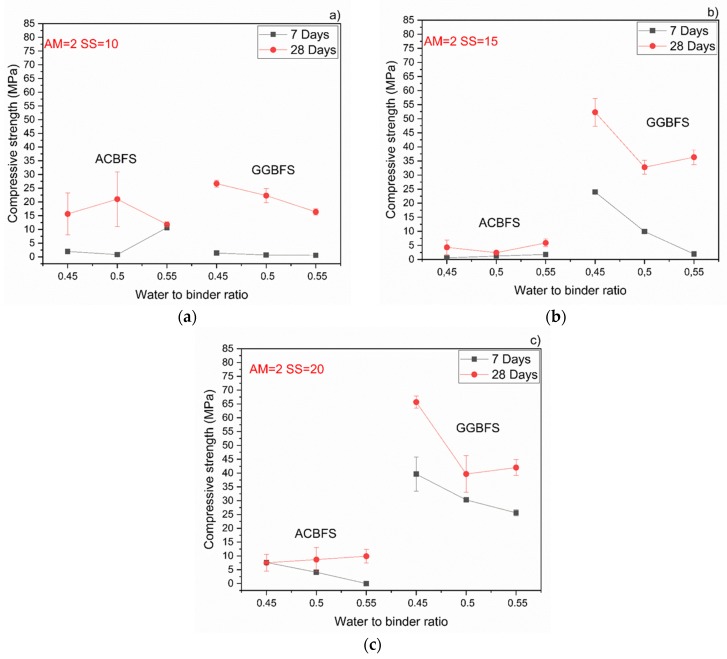
Behavior of the compressive strength evolution with different w/b for MCA-ACBFS and GGBFS sodium silicate activated samples with Ms = 2 and solid solution content. (**a**) 10%; (**b**) 15%; (**c**) 20%.

Both effects were observed also by others and were related to a higher pH values which accelerated dissolution of the slag [24,27,35,36,37]. Higher alkali modulus of the used SS activator decreased the compressive strength values in both slags (Figure 9). In the case of mixes based on the MCA-ACBFS, higher alkali modulus of the SS activator and its higher dosage decreased the compressive strength values from 40 MPa to 20 MPa.

**Figure 10:**
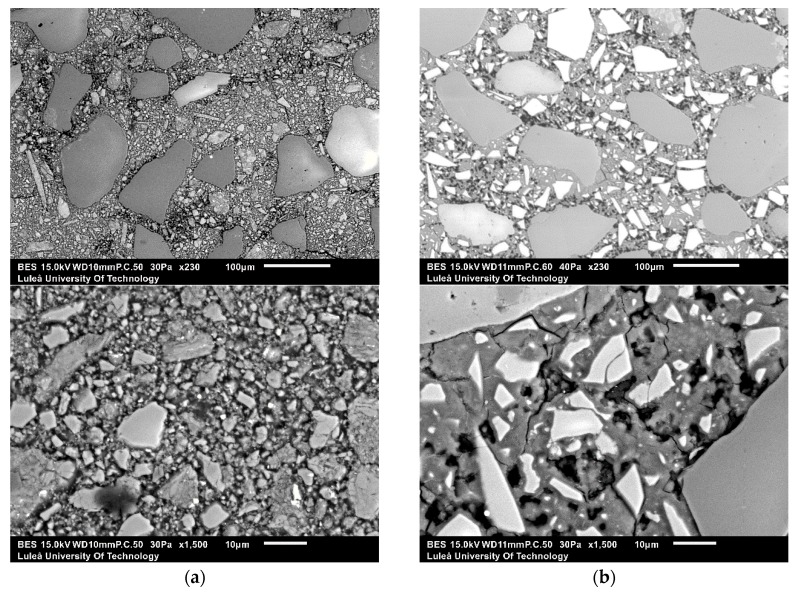
Micrographs of the 28-days old alkali-activated slag samples with w/b = 0.45, AM = 1.5 and SS = 10% using as a precursor (**a**) GGBFS at ×230 and ×1500 of magnification; and (**b**) MCA-ACBFS at ×230 and ×1500 of magnification.

SEM-EDS analysis was performed on alkali-activated slag samples having w/b 0.45, alkali modulus of 1.5 and 10% of solid solution (Figure 10). The alkali-activated GGBFS matrix consisted of homogenous appearing phases surrounding unreacted GGBFS particles and sand. Some air voids and micro cracks were observed as well. On the contrary, the microstructure of the alkali-activated MCA-ACBFS matrix was very inhomogeneous and contained a large amount of fine unreacted MCA-ACBFS particles. These observations complied with the XRD graphs, which shows that after alkali activation the not-amorphized phases have not reacted with the alkali environment (Figure 5).

**Figure 11:**
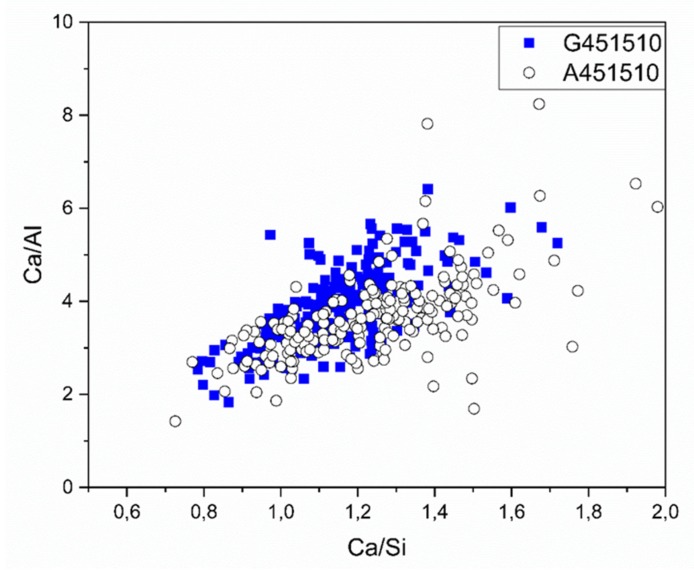
SEM-EDX test results shown as Ca/Al vs Ca/Si ratio collected on 28-days old samples in the binder matrix.

The chemical composition of the solidified binder matrix was characterized by the SEM-EDX spot analysis. The calculated Ca/Al and Ca/Si ratios are shown in Figure 11 and Table 4. The Ca/Si atomic ratio ranged between 0.6 and 1.5, which corresponds to the CASH type phases and also supports the XRD test results (Figure 5).

**Figure 12:**
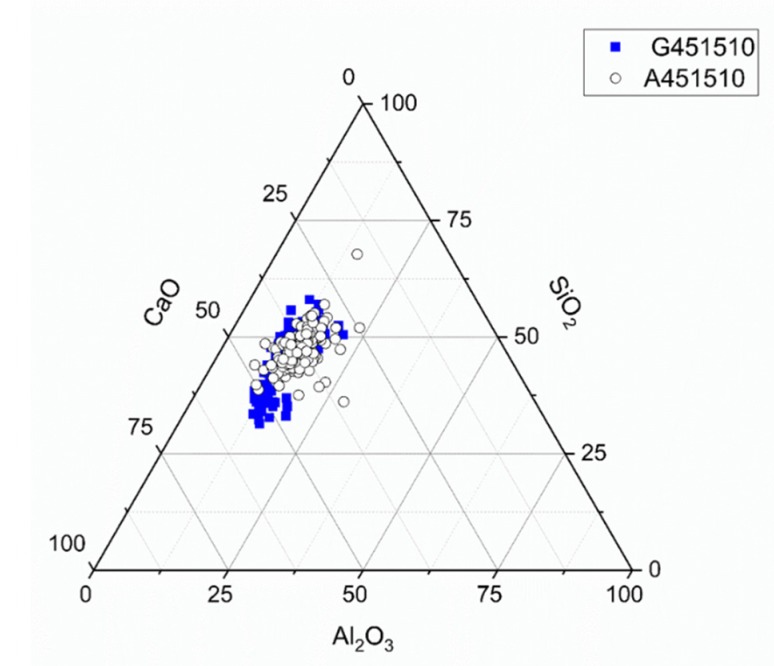
Ternary diagram for sodium silicate activated slags (MCA-ACBFS and GGBFS), with w/b 0.45, alkali modulus 1, after 28 days of hydration.

The ternary diagram (Figure 12) based on the data obtained from the SEM-EDX spot analysis showed a similar distribution for both slags solidified by sodium silicate activation, indicating a dominance of CASH and sodium aluminosilicate hydrate (NASH) [31,38].

## 4. Conclusions

Decreased intensities and increased area of the XRD peaks indicated an increased amorphization of the treated samples after application of the optimized mechanical activation process. SEM-EDX analysis showed a quite similar composition of the solidified binder matrixes in both tested slags. The main conclusions can be summarized as follow:

- Inert and crystalline air-cooled slag can be amorphized and have enhanced reactivity by application of an optimized mechanical activation process.
- MCA-ACBFS can be considered as precursor for alkali activated systems.
- Workability of ACBFS is strongly influenced by the used mechanical treatment and sodium silicate activator.
- The highest compressive strength of mortars based on MCA-ACBFS was achieved for mixes having the w/b = 0.45 and activated with 10% of SS. The AM was 1.
- The highest compressive strength of mortars based on GGBFS was achieved for mixes having the w/b = 0.45 and activated with 10% of SS. The AM was 1.5.

Further tests are needed in order to evaluate the suitability of using MCA-ACBFS as a precursor for AAM. Standardized test methods should be used for full scale comparison with traditional materials.

## Figures and Tables

**Table 1:** Chemical composition of air-cooled blast furnace slag (ACBFS) and ground granulated blast furnace slag (GGBFS) by treatment.

	SiO_2_	Al_2_O_3_	CaO	Fe_2_O_3_	K_2_O	MgO	MnO	Na_2_O	LOI
ACBFS	31.3	11.9	31.1	1.67	0.639	13.7	0.291	0.515	−1.6
GGBFS	34	11.6	30.3	0.291	0.811	12.1	0.516	0.531	−0.9

**Table 2:** Mix composition and sample identification. MCA-ACBFS: Mechanically activated ACBFS.

		MCA-ACBFS			GGBFS		
		Water/Binder			Water/Binder		
Alkali Modulus	SS (wt.%)	0.45	0.5	0.55	0.45	0.5	0.55
1	10	A45110	A5110	A55110	G45110	G5110	G55110
1	15	A45115	A5115	A55115	G45115	G5115	G55115
1	20	A45120	A5120	A55120	G45120	G5120	G55120
1.5	10	A451510	A51510	A551510	G451510	G51510	G551510
1.5	15	A451515	A51515	A551515	G451515	G51515	G551515
1.5	20	A451520	A51520	A551520	G451520	G51520	G551520
2	10	A45210	A5210	A55210	G45210	G5210	G55210
2	15	A45215	A5215	A55215	G45215	G5215	G55215
2	20	A45220	A5220	A55220	G45220	G5220	G55220

**Table 3:** Basicity coefficient and hydration modulus of MCA-ACBFS and GGBFS. HM: Hydration modulus.

	K_b_	HM
MCA-ACBFS	1.10	1.81
GGBFS	0.96	1.58

**Table 4:** Average Ca/Si and Al/Ca atomic ratios calculated based on the SEM-EDX test results.

	Al/Ca	Ca/Si
**AA-GGBFS**	3.87641±0.83344	1.1443±0.16461
**AA-MCA-ACBFS**	3.62735±0.89978	1.22593±0.21916
